# Recreational honey-hunting with honeyguides in the Kingdom of Eswatini

**DOI:** 10.1098/rspb.2025.0255

**Published:** 2025-08-13

**Authors:** Sanele O. Nhlabatsi, Gcina S. Dlamini, Celiwe A. Ngcamphalala, Jessica E. M. van der Wal

**Affiliations:** ^1^ Department of Biological Sciences, University of Eswatini, Kwaluseni, M201, Kingdom of Eswatini; ^2^ Mlindazwe, Shiselweni, Lavumisa, S410, Kingdom of Eswatini; ^3^ Department of Biological Sciences, University of Cape Town, Cape Town, 7701, South Africa; ^4^ FitzPatrick Institute of African Ornithology, University of Cape Town, Cape Town, 7701, South Africa; ^5^ Department of Ornithology, Max Planck Institute for Biological Intelligence, 82319 Seewiesen, Germany

**Keywords:** honey-hunting, greater honeyguide, mutualism, human–wildlife cooperation, globalization, beekeeping

## Abstract

In parts of Africa, people and greater honeyguide birds (*Indicator indicator*) cooperate to access bees’ nests, from which humans harvest honey and honeyguides consume wax. We present the first study of human–honeyguide cooperation in the Kingdom of Eswatini, based on interviews with 83 honey-hunters and beekeepers, and observations of a honey-hunt. We investigated the current extent of honey-hunting with honeyguides, the associated cultural traditions, and the continuity of the practice. We found that honey-hunting with honeyguides is common in Eswatini, primarily as a recreational activity among young boys who herd cattle. Honey-hunters use various acoustic signals to communicate with honeyguides, such as an axe striking wood, verbal praises and whistles—including on hollowed fruit and plastic objects. They reward honeyguides with beeswax, brood and/or honey, fearing future encounters with dangerous animals if they do not. Skills are passed down by elders and spread among young cattle-herders through peer learning. Despite many honey-hunters reporting a decline due to increased education and job opportunities, and habitat loss, the practice is expected to continue. The largely recreational nature of honey-hunting with honeyguides in Eswatini suggests that this interspecies relationship can endure without economic incentives for humans, sustained instead by its cultural and social importance.

­

Please see *AfricanHoneyguides.com/abstract-translations* for a SiSwati translation of the abstract.

## 1. Introduction

In parts of Africa, humans cooperate with greater honeyguide (*Indicator indicator*) birds (hereafter ‘honeyguides’) to locate and harvest bees’ nests, and make their contents accessible for mutual benefit [1]. ‘Honey-hunters’ find and follow a honeyguide to a bees’ nest, after which they subdue the bees and break open the bees’ nest, allowing them to harvest honey, and the honeyguides feed on the leftover wax combs [1,2]. Honey-hunters often make sounds to attract and maintain a honeyguide’s attention, and the honeyguide responds with a chattering call [2]. This rare example of human–wildlife cooperation may predate *Homo sapiens* [3], as honeyguides and honeybees existed in Africa when hominids first started using fire 1.5 million years ago [4], or even earlier, since bees can be subdued by non-fire methods (e.g. [5]). Most empirical studies on human–honeyguide cooperation have focused on eastern Africa (Mozambique [2], Kenya [1,6], Tanzania [5,7,8]), where honey-hunting practices differ among regions and cultures [3,9]. However, this interspecies behaviour likely extends to other regions in sub-Saharan Africa as well [10]. As part of a pan-African research collaborative documenting remaining honey-hunting cultures, we present one of the first detailed accounts of contemporary human–honeyguide cooperation in southern Africa.

The Kingdom of Eswatini (hereafter ‘Eswatini’), a country in southern Africa with rich and diverse flora [11], is well suited for honey collection and production [12]. Yet, despite ongoing initiatives by non-governmental organizations and other agencies to promote beekeeping as an economic development tool [13,14], its adoption remains limited [12,15]. Few emaSwati have taken up beekeeping, and those who do often manage small numbers of hives owing to challenges such as resource constraints and high rates of hive theft [16]. Honey in Eswatini is valued both as a nutritional resource and as a traditional medicine [15,16]; however, there is minimal documentation of wild honey collection [17,18] and the role of honeyguides in locating bees’ nests. Given the prevalence of African honeybees (*Apis mellifera scutellata*), honeyguides [19] and the availability of both natural and managed wooded landscapes [12], human–honeyguide cooperation likely occurs in this country, though it has never before been scientifically documented.

In this study, we examined contemporary honey-hunting practices in Eswatini, with a particular focus on the role of honeyguides in guiding people to bees’ nests. Drawing on structured individual interviews with honey-hunters and several beekeepers, as well as direct observations of a honey-hunt, we investigated: (i) whether and to what extent honey-hunting with honeyguides is practised in Eswatini today; (ii) the practical components of a honey-hunt, including associated cultural practices, that may affect honeyguide behaviour; and (iii) the factors influencing the continuity of this interspecies relationship in this region. By documenting this practice in a new geographical and cultural context, this case study contributes to broader understanding of how human cultural diversity shapes—and is shaped by—cooperative relationships with wildlife, offering insights into the cultural biodiversity of interspecies interactions.

## 2. Methods

### (a) Study area

**Figure 1.**
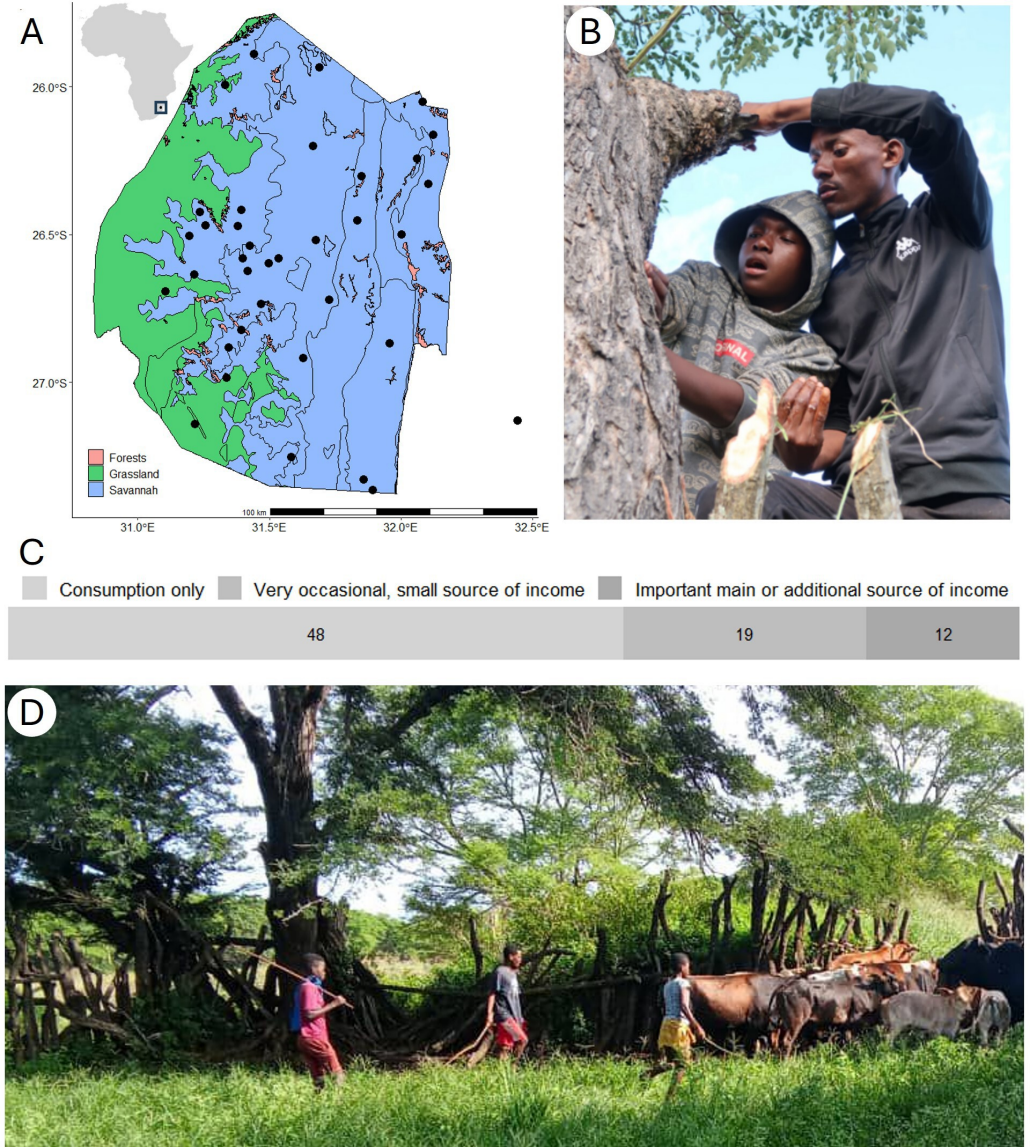
(A) Location and map of Eswatini, with main vegetation types and locations of the 36 communities where the honey-hunter participants reported they honey-hunted. (B) Photo of G.S.D. (right) teaching his nephew how to harvest wild honey. (C) Bar graph showing the predominantly recreational nature of honey-hunting in Eswatini (*n* = 79 honey-hunters). (D) Photo of young Swati cattle herders, the context in which honey-hunting skills are typically learnt. Photo credits: J.E.M.v.d.W. and Muzi Ngcamphalala.

Eswatini (formerly Swaziland) is a small, landlocked country in southern Africa (17 565 km^2^; approx. 175 km from north to south and approx. 130 km from west to east), with a population of approximately 1.2 million people [20]. About 30% of the population resides in urban areas [20], with Manzini, Mbabane and Big Bend being the largest cities. Eswatini is predominantly SiSwati speaking, though Zulu speakers are common along the southern border shared with South Africa’s KwaZulu-Natal province. Eswatini has a sub-tropical climate, with annual rainfall ranging from 500 to 1500 mm and mean annual temperature between 16 and 22°C [21]. The country’s diverse vegetation is influenced by varying altitudes, ranging from lowland savannahs to montane forests (figure 1A). Honeyguides are mostly found in the savannahs and lowland forests [19]. The economy relies heavily on agriculture, with 70% of the population engaging in subsistence farming of crops and livestock [12,15]. Honey-hunting is unrestricted in wild habitats in communal lands but requires permission on private property, and is heavily regulated on plantations owing to wildfire risks, with some areas banning it or requiring scouts to accompany honey-hunters [22].

### (b) Data collection

#### (i) Structured interviews

We conducted structured interviews with 84 honey-hunters and beekeepers during three periods: 17−25 May 2022 (led by S.O.N. and C.A.N., with support of J.E.M.v.d.W. and G.S.D.); 1−17 September 2022 (by S.O.N.) and 11−20 March 2023 (by S.O.N.). We recruited honey-hunters through a radio advert (electronic supplementary material, S1), which resulted in 108 phone calls from potential research participants, some of whom provided further leads for other people to interview. To confirm an interested person’s involvement in honey-hunting, we asked them to explain how honey-hunting with a honeyguide works. We then arranged meetings with those who provided satisfactory answers at locations convenient to them, which was often away from their home communities where they had (previously) honey-hunted owing to migration for work. We also received calls from a few beekeepers who expressed knowledge of honeyguides, leading us to interview several of these beekeepers as well. In all, we interviewed honey-hunters and beekeepers across 23 communities (an umbrella term used for chiefdoms, constituencies, towns and cities), covering honey-hunting activities in 36 communities throughout Eswatini (figure 1A).

At the start of each interview, the interviewer explained the purpose and procedure, and proceeded once the participant had signed a consent form. One participant was under the age of 18, for whom consent was given by an accompanying adult. We conducted interviews mostly in SiSwati, and sometimes in isiZulu, and always in private to prevent participants from influencing each other’s responses. As a thank-you, we gave participants a mobile credit voucher after the interview. Ethical approval for this study was granted by the University of Cape Town Science Faculty Research Ethics Committee (approval no. FSREC 012-2022).

We asked honey-hunters about their experience with honey-hunting, human–honeyguide cooperation, the cultural aspects surrounding these practices, and the changes they perceived in the practice. At the end of each interview, those who reported using calls to communicate with honeyguides were invited to demonstrate them. To assess whether beekeeping poses a threat to the continuation of honey-hunting in Eswatini, we also interviewed 13 honey-hunters who also kept bees, along with three beekeepers from a beekeepers’ cooperative in Ntondozi, to learn about their beekeeping practices and motivations. After excluding one unreliable interview with a honey-hunter, the final sample comprised 83 participants: 80 honey-hunters (77 men, 3 women) and three specialized beekeepers (2 men, 1 woman). For some participants, certain interview questions were omitted owing to interviewer error or time constraints. See electronic supplementary material, S2 for a list of all interview questions analysed and their associated sample sizes.

#### (ii) First-hand observations of a honey-hunt

On 18 May 2022, S.O.N., C.A.N. and J.E.M.v.d.W. accompanied a group of six honey-hunters from Nzomane on a honey-hunt at a nearby private cattle farm. The group included G.S.D. (aged 26, leader), his three nephews (aged 13, 14 and 26), a friend (aged 26) and his brother (aged 23); all but one had been interviewed as part of this study the day before. This group regularly honey-hunts together, providing an opportunity for all authors to directly observe honey-hunting practices involving honeyguides in this area, and to corroborate interview findings.

### (c) Data processing and analysis

We used R version 4.0.2 [23] to analyse and visualize the data. We summarized all categorical data by calculating the percentages for each category. Note that for some questions multiple answers were possible (detailed in electronic supplementary material, S2), in which case the total responses for a given answer did not add up to 100%. Data and code used for analyses are available on OSF (doi:10.17605/OSF.IO/JMFCR) [24].

## 3. Results

### (a) EmaSwati honey-hunting culture with honeyguides

#### (i) Importance of honey-hunting and wild honey

Honey-hunting in Eswatini is a widespread activity among young cattle herders, who eat honey and brood in the wild and often take honey home for parents or other family and neighbours. As adults, many continue honey-hunting in their spare time. When we asked the honey-hunters about their sources of income over the past 12 months, 59% (47 of 80) of honey-hunters reported being employed, 20% (16) reported income from farming or livestock-keeping, 10% (8) from honey sales, 4% (3) from traditional medicine, and 13% (10) reported having no income, including two students and one retiree. Honey-hunting typically occurs in groups, consisting of peers and/or family members of varying ages (electronic supplementary material, S3). Of the 80 honey-hunters interviewed, 80% (64) reported honey-hunting opportunistically while engaging in other activities such as herding livestock, collecting firewood or game-hunting. Among them, 39 honey-hunters also reported embarking on trips specifically for honey-hunting (figure 1B). The remaining 20% (16 of 80) of honey-hunters reported only going on trips dedicated to collecting wild honey, either by locating new bees’ nests or revisiting previously found ones. Honey-hunting trips were consistently reported to be 1 day trips, typically involving minimal travel distance. More detailed statistics on honey-hunting timing and frequency can be found in electronic supplementary material, S3.

Honey-hunters in Eswatini collect wild honey from both honeybees (*Apis mellifera scutellata*), known in SiSwati as ‘tinyosi’ (with honeybee honey referred to as ‘luju’), and stingless bees (unknown species), known in SiSwati as ‘bungolwane’ or ‘bongolwane’*,* along with their honey. Stingless bee honey was reported to be much less common, and was collected by 90% (72 of 80) of honey-hunters, for consumption purposes only. Honey is consumed on its own or used as a natural sweetener in foods and beverages. Additionally, honey is commonly used for medicinal purposes: 83% of honey-hunters (66 of 80) reported using honeybee honey, and 39% (28 of 72) used stingless bee honey for its medicinal properties. Honey is often combined with ingredients like ginger and lemon to create remedies for ailments such as the common cold and ulcers.

Honey harvested from honeybees is mostly used for household consumption only (61%, 48 of 79), with surplus occasionally sold (both in wax combs and in liquid form) to generate a relatively small income (24%; 19 of 79) (figure 1C). Only 15% of honey-hunters (12 of 79) reported significantly relying on honey sales (figure 1C), though this figure is likely overestimating the importance of wild honey sales, as seven also kept bees, and the origin of the honey sold (i.e. wild or from beehives) was not specified. Half of these dedicated sellers sold less than 20 l in the 12 months prior to the interviews, while the other six reported selling between 200 and 400 l, for roughly 1 USD l^−1^ . Honey-hunters reported a wide range of selling units, usually less than a litre, with price variability influenced by factors like location and buyer negotiation (electronic supplementary material, S3). Some honey-hunters (16%; 13 of 80) reported sometimes bartering honeybee honey for other food items, such as maize, chickens, rice and sugar. Honey-hunting efforts reportedly did not increase during years of reduced crop yields.

#### (ii) Honeyguide use and recognition among honey-hunters

The SiSwati name for greater honeyguide is ‘inhlava’, which all honey-hunters interviewed knew or mentioned. Of the 80 honey-hunters, 96% (77 of 80) reported using honeyguides to locate wild bees’ nests. Fifty-eight per cent (44 of 76; 1 participant did not answer this follow-up question) said they only sometimes worked together with honeyguides, 41% (31) said this happened often, while one individual reported always using honeyguides to locate bees’ nests. The three honey-hunters who reported never to have been guided by a honeyguide nonetheless said that they were familiar with the bird. Only one participant reported being guided by a honeyguide to a stingless bees’ nest.

Among the 77 honey-hunters who had been guided by a honeyguide to a bees’ nest, 32% (25) reported having been guided to man-made beehives, and 39% (30) had been guided to other animals: snakes (16), antelopes (15), unspecified wild animals (3), rhinoceros (1) and dead animals (5 cows, 1 human, 1 antelope and 1 unspecified animal). Of these 30 honey-hunters who were guided to destinations other than bees, 90% (27) believed the honeyguide intentionally led them to these other targets, and the other three considered such encounters coincidental while being guided to a bees’ nest. When we asked a subset of 28 honey-hunters who had *not* personally experienced being guided to other things, 57% (16) said they believed that honeyguides are capable of intentionally leading people to other destinations.

Although honey-hunters likely detect and recognize honeyguides largely by their calls, we tested whether honey-hunters could visually identify honeyguides. To do so, we showed honey-hunters that use honeyguides three sets of pictures (in randomized order) of an adult greater honeyguide, a juvenile greater honeyguide and a golden-tailed woodpecker (*Campethera abingoni*) as a control. Eighty-seven per cent (66 of 76) of honey-hunters positively identified the adult honeyguide as a honeyguide, seven did not recognize it, and three mistook it for another bird species. Forty-nine per cent (37 of 76) positively identified the juvenile as a honeyguide, 35 did not recognize it and four mistook it for a different species. Seventy-one per cent (54 of 76) positively identified the golden-tailed woodpecker as a woodpecker (known in SiSwati as ‘inconcodzi’), 18 did not recognize it and four mistook it for a honeyguide.

#### (iii) Learning to honey-hunt from elders and peers

Honey-hunters generally started learning as (young) children: 36% (29 of 80) of honey-hunters reported going on their first honey-hunt between the ages of 5 and 10; 40% (32 of 80) between the ages 11 and 15; 11% (9 of 80) between the ages 16 and 20, and 4% (3 of 80) after turning 21 (electronic supplementary material, figure S2A). The remaining 9% (7 of 80) of honey-hunters could not recall their exact age but stated that they began honey-hunting as young boys.

Ninety-five per cent (75 of 79) of honey-hunters reported having learnt honey-hunting and harvesting skills by watching and being actively taught by others (electronic supplementary material, figure S2B), while the remaining 5% (4 of 79) described themselves as self-taught, having observed others and learnt the practice on their own without formal instruction. Of the 75 honey-hunters who described having learnt socially, 73% (55 of 75) reported learning from peers (friends, brothers, cousins; typically of a similar age or a bit older), 21% (16 of 75) reported learning from elders (parents, grandparents and others from their parents’ generation or older), and 5% (4 of 75) reported learning from both peers and elders. Although 48% (38 of 80) of honey-hunters said that their father (had) honey-hunted, only 18% (7) of those said they learnt the practice from their father, while 16% (6) learnt from a different adult (such as an uncle or grandfather), and the remaining 71% (27) learnt from peers.

When we asked honey-hunters who used calls to communicate with honeyguides (discussed in §3b below) where they had learnt these calls, 33% (25 of 76) said they had learnt them from adults (father, uncles or other elders in the family or community), 62% (47 of 76) had learnt the calls from peers, friends and slightly older boys/brothers during honey-hunting trips, and 5% (4 of 76) said that they came up with the calls on their own. When asked where they had learnt to honey-hunt, 75% (60 of 80) of honey-hunters reported learning in the community in which they were born, and 60% (36 of 60) of those still lived in that community. Of the 25% (20 of 80) who learnt to honey-hunt in a different community from the one where they were born, 45% (9) now live in the community where they learnt, and 15% (3) now live in their place of birth.

### (b) The honey-hunting process

**Figure 2.**
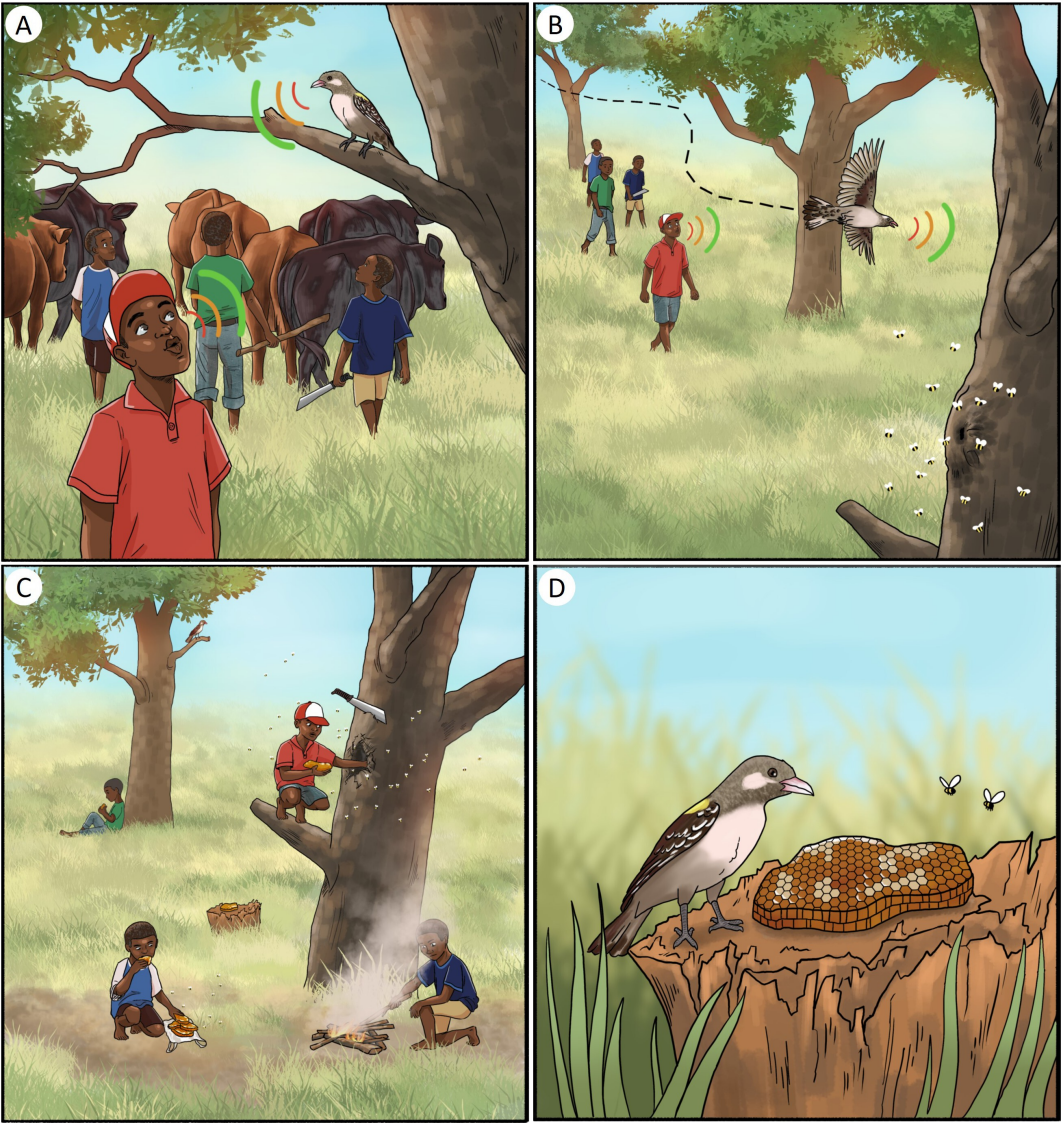
(A-D) The four core steps involved in a typical honeyguide-assisted honey-hunt in Eswatini, based on honey-hunter accounts and first hand observations. (A) Step 1: Initiating cooperation; (B) Step 2: Coordination to a bees’ nest; (C) Step 3: Honey-hunter harvests bees’ nest; (D) Step 4: Honeyguide benefits from the harvest. Illustration by Carissa Gagashi.

Although honey-hunting practices reported varied slightly across individuals and communities, they all followed a core sequence of behaviours, each directly influencing the honeyguide. This section outlines the four core steps of a typical honey-hunt in Eswatini as reported in the interviews (figure 2), and how these were supported by direct observations from a honey-hunt.

#### (i) Four stages of a honey-hunt

#### Step 1: Initiating cooperation

**Figure 3.**
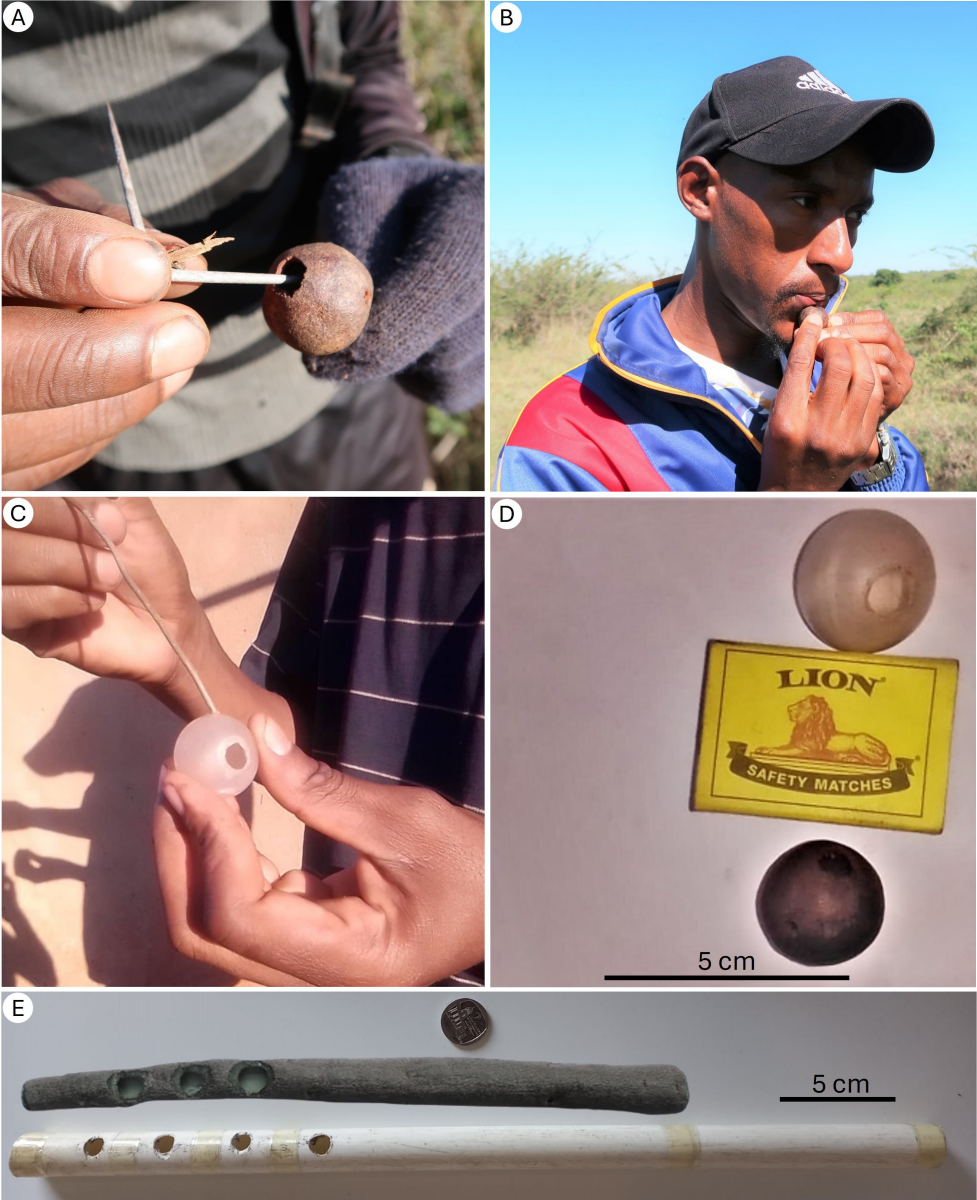
Whistling tools. (A) A honey-hunter uses an acacia thorn to bore two holes and empty Capparis bush seedpod. (B) G.S.D. demonstrates whistling using a *Capparis* fruit whistle (‘ingolgoli’). (C) A honey-hunter burns two holes into a roll-on deodorant ball with a hot iron rod to make a whistle that attracts honeyguides, using it as a substitute when the *Capparis* seedpods are unavailable. (D) Side-by-side comparison of the *Capparis* fruit and plastic ball whistles. (E) Two additional tools used for whistles to attract honeyguides: a hollowed branch with three holes and a PVC pipe with four holes*.*

Human–honeyguide cooperation starts either when a honeyguide approaches the honey-hunter with its chattering call, or when the honey-hunter attracts the honeyguide using sounds (figure 2A). Of the 77 honey-hunters using honeyguides, 70% (54) reported actively using sounds to recruit honeyguides (‘recruitment signals’), 4% (3) reported relying on sounds of human footsteps or loud conversation alone, while the remaining 26% (20) believed the honeyguide need not be summoned, as it would initiate the contact. The most common and widespread recruitment signals were whistles made by mouth (58%; 33 of 57; ranging from non-specific, to specific, melodic tones), whistling on objects (32%; 18 of 57) and an axe striking wood (23%; 13 of 57). Additionally, one honey-hunter reported singing and begging the honeyguide to recruit it, while another described using a quick, rhythmic ‘nt-nt-nt’ clicking sound, made by tapping the tongue against the roof of the mouth, a technique commonly used to attract an animal’s attention. Many honey-hunters employed multiple sounds. In one community, six of eight honey-hunters interviewed reported whistling on dried spherical fruits (seedpods) of *Capparis sepiaria*, hollowed and modified with two holes (locally known as ‘ingolgoli’; figure 3A,B). Two of these honey-hunters reported sometimes also using similarly sized plastic deodorant roll-on balls instead, when the fruit was not available (figure 3C,D). Eleven honey-hunters from five communities in the northeast of the country, separated by up to approximately 50 km, reported using flute-like instruments made from 30 to 40 cm polyvinyl chloride (PVC) pipes with 3 or 4 holes, locally known as ‘umntjingo’ (figure 3E). These PVC flutes were reportedly a new innovation, locally replacing animal horns from impala (*Aepyceros melampus*) and kudu (*Tragelaphus strepsiceros*) and flutes made from plant stems. One honey-hunter still reported using an animal horn, and two brothers used approximately 30 cm crafted flutes with three holes (locally known as ‘lijoye’) from hollow stems of several plants, including *Sclerocarya birrea*, *Ozoroa sphaerocarpa* and *Lannea discolor* (figure 3E). One honey-hunter noted that while a simple whistle sufficed in the past, the scarcity of honeyguides and bees now necessitates specialized whistling tools.

#### Step 2: Coordination to a bees’ nest

The honey-hunter(s) and the honeyguide typically engage in back-and-forth communication as the honeyguide leads the way to the bees’ nest (figure 2B). Out of 77 honey-hunters who use honeyguides, 96% (74) reported actively using sounds to maintain a honeyguide’s attention while following it (‘coordination signals’). Of these, 95% (70 of 74) reported using (softer) whistles, which varied from being described as ‘specific’ or ‘melodic’ whistles learnt from their fathers, to general ‘any’ whistles, cow-motivating whistles, and whistles mimicking the honeyguide’s guiding call. Half of those who used whistles to follow honeyguides reported also using these whistles to communicate with their livestock or domestic animals. Thirty-five per cent (26 of 74) reported saying or singing words, which were described as expressions of praise, endearment, flirting (‘the words would make a girl blush’) or pleading. Example phrases were given of the latter tone: ‘take me there, mama’, ‘show me the honey, I will harvest and save some for you’, ‘I’m with you’ and ‘get me there’. One honey-hunter reported that any sounds or words may be used to communicate with honeyguides, but imitating animal sounds such as bird of prey calls should be avoided, as these may scare the honeyguide away. One honey-hunter reported using the same *Capparis* fruit whistle he used to recruit honeyguides, and another reported clapping his hands. Only 4% (3 of 77) of honey-hunters reported that they did not actively make any sounds while following the honeyguide. Of these, one did not use sounds to attract honeyguides either, while the other two used whistles to attract honeyguides but followed silently. One of them described making a ‘nt-nt-nt’ clicking sound when leaving beeswax as a reward to signal the honeyguide, as he did to recruit honeyguides.

#### Step 3: Honey-hunter harvests bees’ nest

Once the honey-hunters arrive at a bees’ nest, whether located in a tree or in a mound on the ground, they commonly start a fire to produce smoke, which helps subdue the bees (figure 2C). The fire is made from materials like wood, rubber, dry plants or dried cow dung, then extinguished and used to generate smoke directed at the nest entrance. This process is done with care, with 53% (42 of 80) of honey-hunters actively ensuring that the bees and their nest are not exposed to direct flames, which could kill the bees or damage the tree. In addition to smoke, 36% (29 of 80) of honey-hunters reported sometimes using an aromatic herb lemon bush (*Lippia javanica*; ‘umsutane’ in SiSwati). By rubbing its leaves between palms, they produce a strong scent that is believed by some to subdue bees. This method serves as an alternative to smoke when matches are unavailable and, as one honey-hunter noted, prevents the honey from acquiring an undesirable smoky flavour. However, two honey-hunters who did not use this herb argued that it is ineffective, and one suggested that it can anger the bees and thus lead to more stings. Another two honey-hunters explained that they insert a stick into the bees’ nest to check for snakes before harvesting: if bees crawl up the stick, it is taken as an indication that a snake is present.

Honey-hunters then open the nest entrance to remove wax combs using machetes or axes for tree nests, and pickaxes or shovels for ground nests. They mostly eat brood-filled combs as immediate energy on the spot, since eating too much honey causes thirst, and take the honey-filled combs home. Uneaten brood-filled combs are left for honeyguides (detailed in the next step) and/or taken home to make alcoholic beverages (mentioned by 3%; 2 of 80), or to eat. Empty wax combs are sometimes discarded or kept for beekeepers to use as bait in their hives. At home, honey is either eaten in the comb or extracted using force, heat or sieves, then often filtered to remove bits of wax.

Most honey-hunters (73%; 58 of 80) reported leaving behind some wax combs with brood in the nest to keep the bee colony alive for future harvests. Three of these specifically mentioned sparing the ‘heart’ of the bees’ nest, including the queen, to ensure continued survival of the nest. Other measures taken to encourage bees to return include not cutting down trees (60%; 48 of 80) or only cutting when the nest is too high (21%; 17 of 80). Additionally, 69% (55 of 80) indicated they ensure that they open only the smallest possible hole to remove the wax combs, and 91% (73 of 80) reported using bits of wood or rocks to seal the hole afterward.

#### Step 4: Honeyguide benefits from the harvest

Once harvesting is complete, it is customary to reward the honeyguide for its assistance, thus completing the mutualism (figure 2D). All 77 honey-hunters who used honeyguides reported actively rewarding the honeyguide with wax combs with brood (97%; 75 of 77), while 3% (2 of 77) specifically mentioned leaving behind wax combs with honey. The reasons stated for leaving a reward were: to reciprocate the help of the honeyguide (45%; 35 of 77), to help the bird get access to beeswax it otherwise cannot get (32%; 25 of 77), to encourage the honeyguide to guide them to bees again in the future (56%; 43 of 77), and to avoid potentially being guided to a dangerous animal such as a snake (30%; 23 of 77). Two honey-hunters emphasized that beeswax should be left out for honeyguides regardless of whether they guided to the nest. Honey-hunters specified that they leave the wax combs on a nearby tree branch, rock or another convenient place near the harvested nest. Five per cent (4 of 77) reported having occasionally hidden or concealed beeswax from the honeyguide; one individual claimed to do this to punish the honeyguide for ‘not guiding properly’; one mentioned doing this to encourage the honeyguide to lead him to another nest; and the remaining two said they do this for fun, while knowing the honeyguide will eventually find it.

When we specifically asked what would happen if honey-hunters did not leave wax combs for the honeyguide, 79% (61 of 77) indicated that the bird would retaliate by either refusing to lead them to a bees’ nests in the future (10) or leading them to something harmful instead (57). More specifically, 48 honey-hunters reported that failing to reward the honeyguide could result in the bird guiding them—or someone else—to a snake. Of these, five mentioned that the bird might first provoke the snake before guiding them to it. One honey-hunter noted that the honeyguide benefits by leading humans to a snake, as they often flee and leave behind wax combs. Additionally, some honey-hunters mentioned other retaliatory actions, such as leading to a bees’ nest inhabited by a snake (7 responses), an unspecified dangerous animal (4), wild pig (2) and even a human corpse (1). By contrast, one honey-hunter attributed such misfortune to simple bad luck, six believed there would be no consequences for not rewarding the bird, and ten said they were either uncertain or had only heard stories about potential repercussions. Two honey-hunters speculated that the fear of retaliation might be deliberately instilled in young boys as a cautionary tale, ensuring they consistently reward the honeyguide to preserve their mutually beneficial relationship.

#### (ii) First-hand observations from a honey-hunt

Although no honeyguide led the honey-hunting group to a bees’ nest during this trip, we were able to confirm the use of several tools and behaviours described in interviews we did with this group of honey-hunters. The honey-hunters crafted and used *Capparis* fruit whistles, and employed axe-striking against a tree to produce sounds to attract honeyguides. They also used a small fire and smoke from burning rubber to subdue bees. The honey-hunters did not cut down the tree; instead, they widened the entrance hole, left combs in the nest to encourage colony persistence, and sealed the entrance using small rocks. We also observed peer-to-peer skill transmission, as the honey-hunt leader coached his younger nephews through the harvesting process. The honey-hunters ate some of the honey- and brood-filled combs, and took the rest home, and made sure the fire was extinguished before leaving. These observations closely aligned with the information shared during our individual interviews with members of the honey-hunting group. We provide full field notes in electronic supplementary material, S3.

### (c) Continuity and anticipated future of honey-hunting

To evaluate the continuity of honey-hunting with honeyguides, we examined the current activity levels among participants, and their perspectives on changes over time. Among the 80 honey-hunters, 74% (59 of 80) had honey-hunted at least once in the year of, or prior to, the interview (mean ± s.d. age = 39 ± 12; range: 13−66 years), while 26% (21 of 80) had not (mean ± s.d. age = 49 ± 15; range: 28−85 years). These latter ‘inactive’ honey-hunters had last honey-hunted between the ages of 12 and 70 years (mean ± s.d. = 33 ± 16, *n* = 21), and attributed their inactivity to reasons such as relocation to cities for employment, time constraints and honey-hunting inflicted injuries. Just under half (45%; 35 of 77) of honey-hunters reported the practice had changed over their lifetime, citing the following reasons: honey-hunting for income rather than subsistence (10), reduced youth participation (9), declining populations of honeyguides and honeybees (6), unsustainable harvesting (3), and the rise of beekeeping (2). The other 55% (42 of 77) of honey-hunters said that they thought the practice had not changed. When asked about perceived changes in the role of honeyguides in honey-hunting, 77% (58 of 75) said this had not changed, while 23% (17) noted changes. Reasons given for change were lack of interest (56%; 9 of 16) and a decline in participating honeyguides (44%; 7). Of those attributing the change to fewer participating honeyguides, two mentioned that honeyguides were less common, three said that honeyguides no longer call (attributed by one participant to ample food sources besides wax), and two noted that while honeyguides still call, they do so less reliably.

When asked about the anticipated future of human–honeyguide cooperation in Eswatini, 70% (56 of 80) of honey-hunters said they believe that the interaction between humans and honeyguides will continue to exist in the future, of which 26 did not anticipate any changes and 30 thought it would decline. By contrast, 26% (21 of 80) believed the interaction would decline until it ceases to exist, while the remaining 4% (3) were not sure about the continuity of the interaction. The most commonly cited reasons for an anticipated decline in human–honeyguide cooperative interactions were a lack of interest, leading to a loss of knowledge transfer (47%; 24 of 51), and a decline in bee and honeyguide populations, driven by habitat loss and unsustainable honey harvesting practices (43%; 22). Less common reasons given were a breakdown of the mutualistic relationship owing to some people not rewarding the honeyguides and then honeyguides failing to guide to bees (16%; 8 of 51), the rise of beekeeping (8%; 4), the direct killing of honeyguides for use in magic potions (8%; 4), and restricted access to land that supports sufficient populations of both honeyguides and bees (6%; 3).

To understand whether beekeeping is a credible threat to the continuation of honey-hunting in Eswatini, we interviewed a total of 15 beekeepers to understand their beekeeping practices and motivations: three specialized beekeepers from a beekeeping cooperative, and 12 honey-hunters who also kept bees. Most beekeepers (93%; 14 of 15) used box hives, while one participant also reported using solid wood cylinder hives, and one participant reported keeping bees in a tractor tyre. The majority (67%; 10 of 15) of participants had 10 or fewer hives, 20% (3) had 11−20 hives and 13% (2) had over 20. When harvesting, 100% of beekeepers reported taking out only wax comb with honey, leaving behind empty and brood-filled combs. Unlike honey-hunters, of whom only 24% (19 of 80) reported occasionally selling their surplus (see above), 100% (15 of 15) of beekeepers cited selling honey as their main motivation. Over half (53%; 8 of 15) reported also using honeybees for food and medicine. When we asked how they learnt beekeeping, 53% (8 of 15) of beekeepers had taken courses from organizations (World Vision, Bulembu Group, Ministry of Agriculture, Eswatini Water and Agricultural Development Enterprise); 27% (4 of 15) had learnt from a family member (i.e. father, brother-in-law or sister-in-law), and 20% (3 of 15) were taught by another experienced beekeeper in their area. Only 20% (3 of 15) of beekeepers reported having a parent or grandparent who kept bees. All three specialized beekeepers reported that they did not honey-hunt because they found beekeeping easier and safer, and two specified the danger of snakes in forests. When comparing honey quality, 75% (6 of 8) of beekeepers believed beekeeping produced more honey of similar quality to honey-hunting, while the remaining beekeepers felt beekeeping resulted in better quality honey. When asked about the future of honey-hunting with honeyguides in Eswatini, two specialized beekeepers believed that the practice would be replaced by beekeeping and eventually cease, and the other thought the practice might decline but would continue to some extent.

## 4. Discussion

Honeyguides likely play an important role in many honey-hunting cultures across sub-Saharan Africa. However, this behaviour, and the extent of its variation across different regions and cultures, remains under-documented. As part of a pan-African research collaborative to record remaining honey-hunting cultures, we present the first documentation to our knowledge of human–honeyguide cooperation in Eswatini, and one of the first case studies in southern Africa. Honey-hunting with honeyguides is widely practised in Eswatini, primarily as a recreational activity among young boys herding cattle (figure 1D). Brood and small amounts of honey are typically consumed on the spot for an instant energy boost, while the remainder is taken home to share with family and neighbours. These findings contribute to the growing documentation of the role of honeyguides in African honey-hunting practices, and expand our understanding of the cultural diversity shaping human–wildlife cooperation.

Honey-hunting in Eswatini is primarily a ‘side’ activity done in people's free time from their jobs or livelihoods, and wild honey is rarely sold. This contrasts with other honey-hunting cultures, such as the Hadzabe in northern Tanzania [8], the Yao in northern Mozambique [2,25] and the Awer in Kenya [6], where honey-hunting plays a significant economic role, supporting livelihoods and contributing to local economies. This difference may stem from several factors, including the subsistence economies of the regions (the Hadzabe and Awer are traditionally hunter–gatherer societies, the Yao follow mixed subsistence, while the emaSwati are primarily pastoralists and farmers), Eswatini’s relatively urbanized environment and higher economic status, and a well established sugar industry—Eswatini is the third-largest producer of cane sugar in Africa [26]. The largely recreational nature of honey-hunting with honeyguides in Eswatini demonstrates how this interspecies relationship can persist even without economic drivers for humans, highlighting its enduring cultural and social significance. It offers individuals, particularly young boys, opportunities to connect with their natural environment, develop practical bush skills, and participate in a shared cultural tradition.

Honey-hunting with honeyguides is widespread across Eswatini, and a common skill among young boys in rural areas, unlike in other cultures where it is typically restricted to a small subset of the population [2]. Moreover, within Eswatini’s compact landscape (approximately 175 km from north to south and approximately 130 km from west to east), there is considerable variation in the honey-hunting signals used. This widespread nature of the practice and regional variation in signals are likely influenced by two interconnected factors. First, learning is reported to occur not only through elders like in other honey-hunting cultures [2,8], but also significantly through peer interactions, promoting wider access to the practice and enabling a greater diversity of learning models. This dual learning pathway, in turn, can foster greater variation in honey-hunting skills and cultural variants, and consequently, influence partner quality from the honeyguide’s perspective. A similar pattern has been observed in human–dolphin cooperation, where fishers who acquired their skills though vertical transmission were found to be more effective cooperative partners [27]. Additionally, the economic importance of honey-hunting likely shapes the behaviour of those engaged in the practice and the pressure placed on them to cooperate efficiently and maximize returns. Social or economic pressure to gain as much honey as possible per unit time, or lack thereof, may either reinforce or reduce pressure on individuals to adopt group-level behaviours that enhance coordination and success [28]. In regions like northern Mozambique, for example, where honey-hunting is more economically and subsistence-driven, and skills are mostly passed down vertically, honey-hunters have converged on a single, highly effective call [2,25]. By contrast, the greater variation in honeyguide-related traits in Eswatini likely in part reflects the recreational value of the practice, with less urgent pressures for efficient cooperation. Despite the variation in honey-hunting behaviours across the country, the continued success of honey-hunting with honeyguides suggests they either flexibly respond to different human sounds or can learn and adapt to a wide range of culturally specific signals [9].

The diversity in honey-hunting signals also reflects broader changes in honey-hunting traditions. In areas where objects are used to whistle and attract honeyguides, some honey-hunters sometimes now opt for easily accessible plastic materials instead of fruits and plant stems. This shift highlights the adaptive nature of cultural practices, demonstrating how synthetic materials are incorporated into longstanding traditions [29], supporting the continued evolution and resilience of honey-hunting in contemporary contexts.

An important aspect of many honey-hunting traditions is the emphasis on rewarding the honeyguide for its help, along with the feared consequences of failing to do so [30]. Swati honey-hunters routinely leave wax combs for honeyguides, similar to, for example, the Yao honey-hunters [2]. Eighty per cent of participants in this study shared a belief that honeyguides retaliate when unrewarded, by leading humans to snakes and other dangerous animals, a behaviour reported in many other honey-hunting cultures across Africa [30], and personally experienced by several participants and an author (G.S.D.) of this study. Some honey-hunters suggested that this fear may be intentionally passed down to young boys as a cautionary tale, ensuring that they always reward the honeyguide and thus maintain the cooperation. Given that children’s ability to cooperate is shaped by cultural teachings on appropriate behaviour [31], this story—whether true or not—illustrates how oral traditions and teachings can reinforce respect and care for animals on which people rely [32], and in this case potentially sustaining mutualistic interactions across generations. More broadly, the belief in the honeyguide’s agency, combined with the rarity of harm toward the bird in Eswatini, suggests a broader cultural attitude of respect that may contribute to its protection.

Swati honey-hunters use several methods to ensure that bees’ nests remain active after harvest, such as leaving some brood-filled combs in the nest and sealing the bees’ nest entrances post-harvest, practices also described in some other cultures (e.g. [5,8]). Lemon bush is sometimes used as an alternative to smoke (also see[33]) but not specifically to maintain bee colonies, although fire alternatives in other honey-hunting cultures have been considered to be less disruptive to bee colonies [5]. Further research is needed to assess the effectiveness of these measures in encouraging bees to stay, their impact on overall bee populations, and whether these behaviours make honey-hunting more sustainable compared with regions where such practices are not employed (e.g. [2]). These practices also likely affect honeyguides. On one hand, by helping maintain the bee colonies, these actions might encourage honeyguides to continue cooperating with humans in the future, possibly ensuring a more sustainable and long-term mutualism. On the other hand, these practices might reduce the immediate benefits for honeyguides, as the bees may still be present or active, making it riskier for the birds to access the wax. Additionally, ensuring that a harvested nest remains active for future use may reduce the reliance on honeyguides to find new nests.

When asked about the continuity of human–honeyguide cooperation, honey-hunters believed that it will most likely continue, though many expressed concerns that it has already declined and may continue to do so. Reported drivers of this decline included reductions in honeybee and honeyguide populations owing to deforestation and drought, restricted access to commercial forests and protected areas, and decreasing youth participation as a result of education and work commitments. Similar concerns have been reported in other regions, where the erosion of honey-hunting with honeyguides has been linked to environmental degradation, forest access limitations, and generational disengagement [5–7,10,34]. However, unlike in areas where the practice is largely restricted to elders and thus at greater risk of being lost [6,34], in Eswatini it remains known and practised by younger generations, suggesting that as long as people continue to live pastoralist lifestyles, the practice is unlikely to disappear entirely. Moreover, while beekeeping has been identified as a considerable threat to human–honeyguide cooperation in some countries [5,7,35], in Eswatini it remains relatively small-scale and does not seem to threaten the interaction. How the (lack of) economic incentive for honey-hunting with honeyguides, in this region or any other, shapes who participates and influences its continuation, requires further study.

## 5. Conclusion

In Eswatini, despite globalization and the increasing availability of commercial honey and sugar, honey-hunting remains an important recreational activity, primarily practised by young boys in rural areas. This tradition reflects a dynamic and evolving practice, characterized by variations in communication methods, the integration of modern materials into traditional tools, and the use of sustainable practices aimed at preserving bee populations. Although challenges such as declining bee populations and reduced youth engagement have been reported, the social and cultural significance of honey-hunting suggests it will likely endure as a part of Eswatini’s cultural heritage. This case from Eswatini offers new insights into how a culturally embedded and largely recreational form of human–wildlife cooperation can be sustained without economic incentives. More broadly, it highlights how positive cultural traditions can foster resilient and adaptive forms of coexistence with wildlife.

## Data Availability

Anonymized interview data are available on the Open Science Framework (OSF) [24]. Supplementary material is available online [36].

## Dedication

We dedicate this paper to the late Dominic Bhekimpi Ngcamphalala, C.A.N.’s father, whose 'tales' of honey-hunting with honeyguides as a young cattle herder in Eswatini made C.A.N. roll her eyes—but now we know those stories to be true.
